# Supplementary material for: Mutations in PCYT2 disrupt etherlipid biosynthesis and cause a complex hereditary spastic paraplegia
Source: Brain. 2019 Oct 22;142(11):3382–97. doi: 10.1093/brain/awz291 (PMC6821184; doi:10.1093/brain/awz291)
Supplement: awz291_Supplementary_Data [file awz291_supplementary_data.zip › awz291-Suppl_data/Supplementary_Data4.zip!/Supplementary information 3 - fibroblast lipidomics.zip!/Fibroblast lipidomics/Controle vs Patient2/Statistical plots and data/PS species/Volcano plot_alternative.pdf]

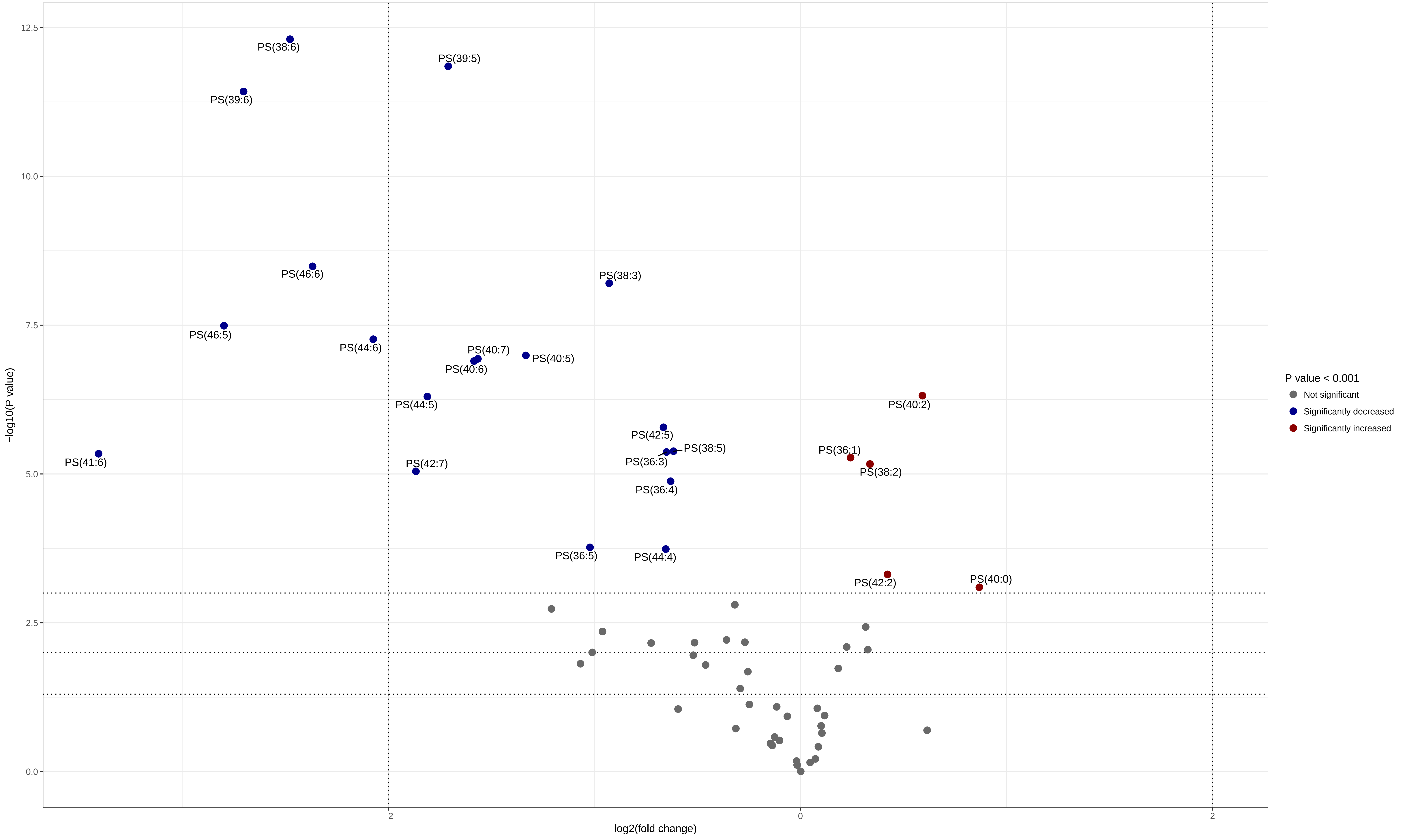

PS(38:6)
PS(39:5)
PS(39:6)
PS(46:6)
PS(38:3)
PS(46:5)
PS(44:6)
PS(40:7)
PS(40:5)
PS(40:6)
PS(44:5)
PS(40:2)
PS(42:5)
PS(38:5)
PS(36:1)
PS(36:3)
PS(41:6)
PS(42:7)
PS(38:2)
PS(36:4)
PS(36:5)
PS(44:4)
PS(42:2)
PS(40:0)
P value < 0.001
Not significant
Significantly decreased
Significantly increased
−log10(P value)
log2(fold change)
12.5
10.0
7.5
5.0
2.5
0.0
−2
0
2
